# Preparation and Performance Evaluation of a High Temperature Stable Magnetorheological Fluid with Shear-Thinning Resistance

**DOI:** 10.3390/ma18163840

**Published:** 2025-08-15

**Authors:** Xiangfan Wu, Yangyang Guo, Zuzhi Tian, Haopeng Li, Zhiyuan Shi

**Affiliations:** 1 School of Mechanical and Electrical Engineering, Xuzhou University of Technology, Xuzhou 221018, China; 2 School of Mechanical and Electrical Engineering, China University of Mining and Technology, Xuzhou 221116, China; 3 State Key Laboratory of Intelligent Mining Equipment Technology, Xuzhou 221116, China; 4 China Mining Products Safety Approval and Certification Center, Beijing 100028, China

**Keywords:** magnetorheological fluid, thermal stability, shear-thinning, sedimentation rate, shear yield stress

## Abstract

Magnetorheological fluid exhibits shear-thinning behavior when subjected to high temperature environments exceeding 100 °C, which will significantly compromise the operational stability and reliability of the associated mechanical systems. To enhance the performance of magnetorheological fluid, this study selects soft magnetic particles, base carrier fluid, and surfactants based on their resistance to high temperatures and shear-thinning effects. A novel magnetorheological fluid with enhanced thermal stability and shear stability is subsequently developed by carefully selecting flake-shaped carbonyl iron powder, dimethyl silicone oil, and surfactant exhibiting both sedimentation stability and high temperature resistance. The apparent rheological properties and mechanical performance of the fluid are systematically evaluated. Experimental results indicate that the sedimentation rate of the prepared magnetorheological fluid is 3.86% after standing for 10 days, the thermal expansion rate at 200 °C is 12.8%, and the evaporation rate following repeated high temperature applications is only 0.66%. The shear yield stress of the prepared magnetorheological fluid is 31.2 kPa under the magnetic field of 817 mT. The prepared magnetorheological fluid demonstrates excellent thermal stability and shear-thinning resistance, which holds significant potential for enhancing the performance of magnetorheological devices in future applications.

## 1. Introduction

Magnetorheological (MR) fluid is a type of intelligent material, which is composed of soft magnetic particles, base carrier fluid, and surfactants [1]. In the absence of an external magnetic field, soft magnetic particles are uniformly dispersed within the base carrier fluid, allowing the liquid to flow freely. Under an applied magnetic field, the reversible MR effect is activated as the particles align into chain-like structures, resulting in a solidification behavior characterized by tunable yield stress [2,3].

MR fluids have been widely applied in vibration control fields such as vehicle suspensions [4,5,6], medical equipment [7], civil buildings [8], aerospace vehicles [9], and weaponry [10], owing to their advantages of intelligent controllability, low energy consumption, and rapid response [11]. In addition, these fluids are also utilized in power transmission [12], polishing [13], sealing [14], and other related applications.

However, in practical applications, MR fluids face challenges such as particle sedimentation, decreased thermal stability, and shear-thinning behavior, which have hindered the widespread adoption of magnetorheological devices. Consequently, numerous researchers are actively investigating strategies to enhance the performance of MR fluids. The commonly used methods to improve the properties of MR fluids can be classified into the following aspects:

(a) Increasing the particle concentration of MR fluid. Zhang et al. [15] found through experiments that under the same magnetic field intensity, the shear yield stress of MR fluid with a soft magnetic particle mass fraction of 60 wt% was 2.32 times higher than that of MR fluid with a mass fraction of 40 wt%. (b) Exploring soft magnetic materials with high permeability and saturation magnetization. Anupama et al. [16] prepared nickel-zinc-iron oxide (Ni_0.5_Zn_0.5_Fe_2_O_4_) powder by solution combustion method. Ni_0.5_Zn_0.5_Fe_2_O_4_ has a higher saturation magnetization, and the shear yield stress of MR fluid is effectively improved by adding Ni_0.5_Zn_0.5_Fe_2_O_4_ powder. (c) Mixing larger and smaller soft magnetic particles. Laherisheth et al. [17] prepared MR fluid by adding nanoparticles to general particles, finding that the microstructure of MR fluid was enhanced by the addition of nanoparticles. (d) Selecting particles with rough surfaces or irregular shapes to increase the friction between particles. Kwon et al. [18] synthesized the octahedral magnetite particles by the auto scope hydrothermal method and added them to the MR fluid whose mass fraction was 70 wt%. Results show that the viscosity, yield stress of the MR fluid are effectively improved. (e) Improving the performance of the base carrier fluid (viscosity, fluidity, and stability) or finding a novel base carrier fluid. Kumar et al. [19] used vegetable oil (coconut oil) as the base carrier fluid to prepare MR fluid, which had a more robust structure under higher magnetic field intensity. Dorosti et al. [20] prepared a new water-based MR fluid using wormlike micelles, showing better sedimentation stability and yield stress. (f) Exploring different surfactants and additives to improve stability and rheological properties. Yari et al. [21] used a lecithin/cyclohexane/water system enabled the formation of stable WLMs, allowing MR fluid with 70 wt% carbonyl iron particles to achieve a notably low sedimentation rate of 5.3% after one week-significantly improving upon previous formulations.

The application of one or more of the aforementioned methods can enhance the properties of MR fluids. However, these approaches are primarily based on room-temperature conditions. In practical operation, MR fluids inevitably experience elevated temperatures due to heat generated by energized coils and inter-particle friction. Arief found that high temperatures will cause the thinning effect of MR fluids [22]. Rabbani investigated the influence of temperature on the stability of MR fluids through experiments, finding that a temperature increase will lead to serious sedimentation [23]. High-temperature environments can cause thermal expansion and sedimentation in MR fluids, leading to a degradation of their mechanical properties, which ultimately compromises the performance and reliability of MR devices.

Besides, MR fluids are typical non-Newtonian fluids with a shear-thinning effect. Dai et al. [24] investigated the shear-thinning law of MR grease with different particle concentrations, finding that the shear-thinning effects become more obvious with the increase of particle concentrations. Wang et al. [25] found that the shear-thinning behavior of MR fluids is positively linear with concentrations and viscosity of silicone oil. Pop et al. [26] concluded that the increase in shear rate will lead to shear thinning, which is closely related to the microstructure of MR fluids. When the shear rates increase, the scattered intensity and anisotropy of particle chains grow, leading to the destruction of particle chains and eventually shear-thinning effects. Felicia et al. [27] also attributed shear-thinning to the destruction of particle chains. The previous work has confirmed that the shear-thinning effect of MR fluid will reduce the accuracy of the constitutive model, which will adversely affect the precise control of MR devices.

Building on previous research [28,29], this study aims to develop a novel MR fluid with enhanced resistance to high temperatures and shear-thinning effects. Soft magnetic particles, base carrier fluid, and surfactants are individually evaluated and selected based on their thermal and shear stability performance to identify suitable materials for each of the three primary components of MR fluid. Based on these selections, a novel MR fluid with improved high-temperature and shear-thinning resistance is formulated, and its apparent and mechanical properties are systematically assessed. The prepared magnetorheological fluid exhibits excellent thermal and shear stability, demonstrating promising potential for enabling stable and reliable operation of magnetorheological devices.

## 2. Selection of Materials

The shear-thinning behavior of MR fluids under a magnetic field arises from the reduction in the number of soft magnetic particles that rotate in synchrony with the driving disks due to the influence of the Navier-Stokes force [30]. Therefore, it is crucial to prevent the detachment of soft magnetic particles from the driving components and moderately enhance the viscosity of MR fluids during the preparation of MR fluids with improved resistance to high temperature and shear-thinning. Based on the interaction between the soft magnetic particles and the base carrier fluid, the following strategies are proposed to increase the viscosity of MR fluids in this paper: (a) Enhance the interparticle forces to counteract the particle detachment induced by the Navier-Stokes force; (b) Select a base carrier fluid with an appropriate viscosity.

In the absence of an external magnetic field, density differences between the soft magnetic particles and the base carrier fluid may lead to sedimentation. Hence, the use of surfactants is recommended to ensure good sedimentation stability of the MR fluid. Moreover, the selected materials should exhibit thermal stability at elevated temperatures, as MR fluids often operate under high-temperature conditions caused by coil energization and inter-particle friction.

### 2.1. Soft Magnetic Particles

**Figure 1:**
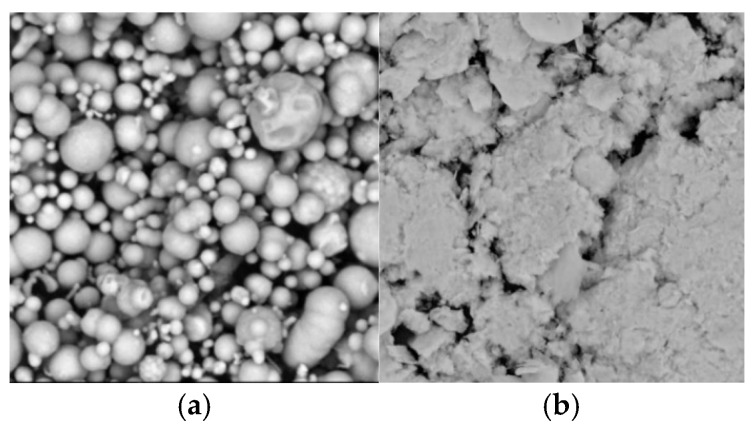
SEM photos of CIPs in different shapes: (**a**) ball shape (1μm); (**b**) flake shape (10 μm).

Soft magnetic particles are the components with the highest mass fraction in MR fluids and serve as the primary source of shear yield stress during the MR effect. Materials exhibiting high magnetic permeability and thermal stability are typically selected for this role. Currently, carbonyl iron powder (CIP) with varying morphologies is the most widely used type of soft magnetic particle. Figure 1 presents the SEM images of CIP. And the specific parameters of the CIP used are summarized in Table 1.

### 2.2. Surfactants

Surfactants are employed to achieve good sedimentation stability in MR fluids by preventing soft magnetic particles from settling. These surfactants possess short molecular chains at the microscale, with one end attached to the particle surface and the other extending outward to inhibit direct particle-to-particle contact. In previous studies, surfactants have been classified into four types: anionic, cationic, zwitterionic, and non-ionic surfactants. Furthermore, the orthogonal test method was applied to blend surfactants with coupling agents and thixotropic agents, confirming that the following two surfactant combinations can effectively enhance the sedimentation stability of MR fluids [28].

Combination A consists of 2.0 wt% water-soluble imidazoline, 2.0 wt% glycol monostearate, 2.0 wt% glycerol monostearate, 1.0 wt% silane thixotropic agent, and 1.0 wt% hydrophobic fumed silica thixotropic agent. Experimental results indicate that the viscosity of the MR fluid prepared using Combination A is 1.02 Pa·s at room temperature, with a sedimentation rate of 6.12% after one week of storage. Table 2 provides detailed information on the reagents used in Combination A.

Combination B consists of 1.5 wt% sodium lauryl phosphate, 1.0 wt% sodium fatty acid methyl ester sulfonate (compound MES), 1.5 wt% glycol monostearate, 1.5 wt% glycerol monostearate, and 1.0 wt% hydrophobic fumed silica thixotropic agent. Experimental results indicate that the viscosity of the MR fluid prepared using Combination B is 1.09 Pa·s at room temperature, with a sedimentation rate of 1.61% after one week of storage. Table 3 provides detailed information on the reagents used in Combination B.

The novel MR fluid with high-temperature and shear-thinning resistance is expected to remain in static placement for extended periods when utilized in MR devices, thus necessitating good sedimentation stability. Further studies will be conducted to evaluate the stability of the MR fluids prepared using combinations A and B.

### 2.3. Base Carrier Fluid

The base carrier fluid significantly influences the properties of MR fluids, including tribological behavior, sedimentation stability, and thermal stability. MR fluids are typically categorized into water-based and oil-based types, with water and oil serving as the respective base carrier fluids. During operation, MR fluids in MR devices are inevitably exposed to high-temperature environments for short periods due to coil energization and friction between components, with temperatures occasionally exceeding 100 °C. Under such conditions, the water in water-based MR fluids may boil and evaporate, leading to instability in the fluid’s performance. Moreover, oxidation can occur when water, soft magnetic particles, and air come into contact, resulting in corrosion of the MR fluid. Compared to water-based MR fluids, oil-based MR fluids exhibit a smaller density difference between the base carrier fluid and the soft magnetic particles, which helps suppress sedimentation. Therefore, MR fluids with enhanced resistance to high temperature and shear-thinning are typically formulated using oil-based carrier fluids.

Dimethyl silicone oil is the most widely used base carrier fluid for MR fluids [31]. Hydroxyl silicone oil and polyethylene glycol 300 have also been employed as base carrier fluids in other MR suspensions [32]. In industrial applications, high-temperature heat transfer oils, gear oils for overload conditions, vacuum pump oils, and perfluoropolyether lubricants are commonly utilized under elevated temperature conditions due to their excellent thermal stability. With the increasing emphasis on green and sustainable development, natural oils such as sunflower oil, cottonseed oil, olive oil, simarouba oil, and mahua oil have been introduced as alternative base carrier fluids for MR fluids, contributing to improved performance characteristics [33,34,35]. This study aims to systematically evaluate the high-temperature and shear-thinning behaviors of three selected oil types and subsequently develop an MR fluid with enhanced resistance to high temperature and shear-thinning by selecting the optimal base carrier fluid. Table 4 presents detailed information on the three types of base carrier fluids intended for preparing the MR fluid with high-temperature and shear-thinning resistance.

Among the base carrier fluids listed in Table 4, coconut oil and palm oil are solid at room temperature, whereas the remaining nine types of oils remain in liquid form under the same conditions. High-load gear oil appears dark brown, vacuum pump oil is light brown, olive oil is dark brown, and palm oil exhibits a light yellow color, while the other seven types of oils are colorless and transparent.

## 3. Testing of Material Properties

### 3.1. Soft Magnetic Particles

(1) Magnetic permeability

The MR effect relies on the magnetization of particles under an applied magnetic field, making magnetic permeability a critical property for soft magnetic particles. Previous research [29] has demonstrated that CIP exhibits no significant reduction in permeability or shear yield stress at temperatures up to 200 °C, indicating excellent thermal stability. Therefore, CIP is suitable as a soft magnetic particle material for MR fluids with high-temperature and shear-thinning resistance.

(2) Shear-thinning resistance

**Figure 2:**
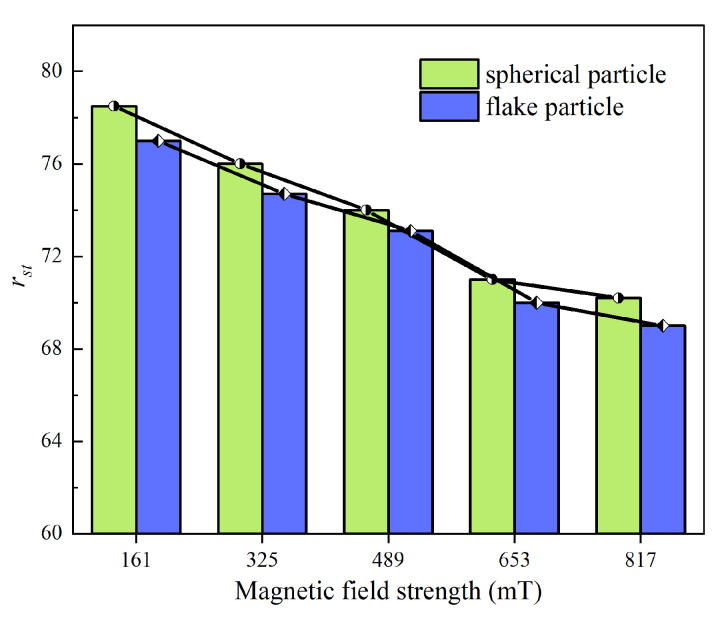
Comparison of shear-thinning effects of MR fluids prepared with different CIPs.

To evaluate the shear-thinning resistance, two types of MR fluids with a mass fraction of 60 wt% (18.3 vol%) were prepared by dispersing spherical and flake-shaped CIPs in dimethyl silicone oil, respectively. The rotational rheometer MCR 302 equipped with an MRD 170/1 T MR module was employed to measure the viscosity across a shear rate range from 0.01 s^−1^ to 100 s^−1^. The tests were conducted at room temperature. The apparent viscosity of each MR fluid was recorded under magnetic field strengths of 161 mT, 325 mT, 489 mT, 653 mT, and 817 mT. The shear-thinning behavior was quantified using the ratio *r*_s_ of maximum apparent viscosity *η*_max_ (0.01 s^−1^) to minimum apparent viscosity *η*_min_ (100 s^−1^), as defined in Equation (1). Figure 2 compares the shear-thinning performance of MR fluids formulated with CIPs of different morphologies.

(1) $$r_{s}=\frac{\eta_{\max}}{\eta_{\min}}$$

As shown in Figure 2, the shear-thinning effect of MR fluids prepared using spherical and flake-shaped particles decreases with increasing magnetic field strength. This occurs because a stronger magnetic field generates greater interparticle forces, which prevent the particles from dislodging. Under the same magnetic field conditions, the *r*_s_ value of flake-shaped particles is lower than that of spherical particles, indicating that MR fluids formulated with flake particles exhibit higher resistance to shear-thinning. The particle chains formed by flake-shaped particles are more tightly packed under the magnetic field, making it more difficult for the particles to detach when the chain rotates with the shear disk.

(3) Shear yield stress

The testing temperature was maintained at room temperature by the circulating water within the module. The yield stress of the MR fluid under each magnetic field strength (61 mT, 325 mT, 489 mT, 653 mT, and 817 mT) was subsequently measured and recorded.

**Figure 3:**
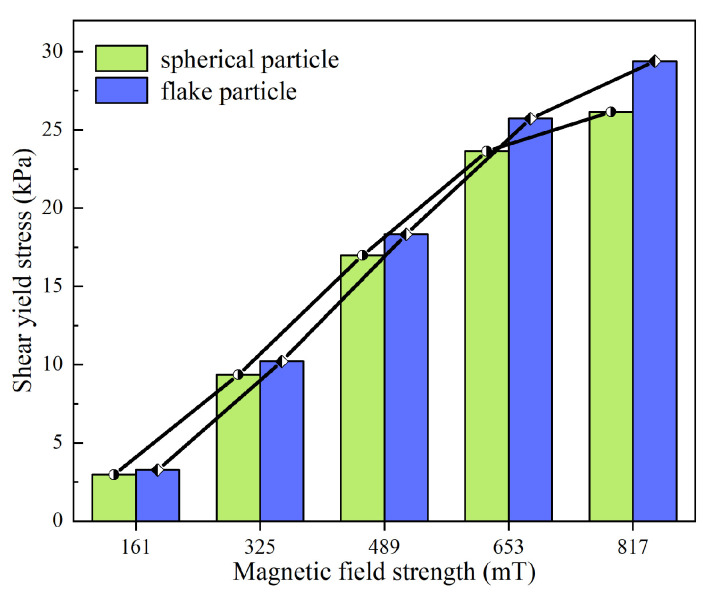
Comparison of the shear yield stress of MR fluids prepared with different CIPs.

As shown in Figure 3, the shear yield stress of MR fluids prepared using flake-shaped and spherical particles increases with the strengthening of the magnetic field; however, the rate of increase gradually diminishes. The MR fluid formulated with flake-shaped particles consistently exhibits a higher shear yield stress compared to that with spherical particles across all tested magnetic field strengths, and the disparity becomes more pronounced as the field strength increases. Under the same magnetic field, the particle chains formed by flake-shaped particles are more closely aligned than those formed by spherical particles [36], resulting in stronger interparticle forces that enhance resistance to chain disruption under shear.

In summary, flake-shaped CIPs demonstrate excellent thermal stability, superior shear-thinning resistance, and higher shear yield stress, making them well-suited for use as soft magnetic particles in MR fluids designed for high-temperature and shear-thinning environments.

### 3.2. Surfactants

**Figure 4:**
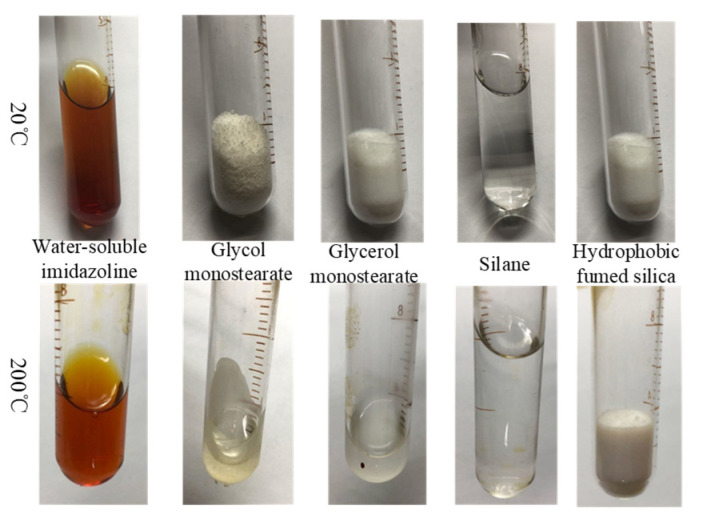
Comparison of apparent properties of surfactants combination A at 20 °C and 200 °C.

**Figure 5:**
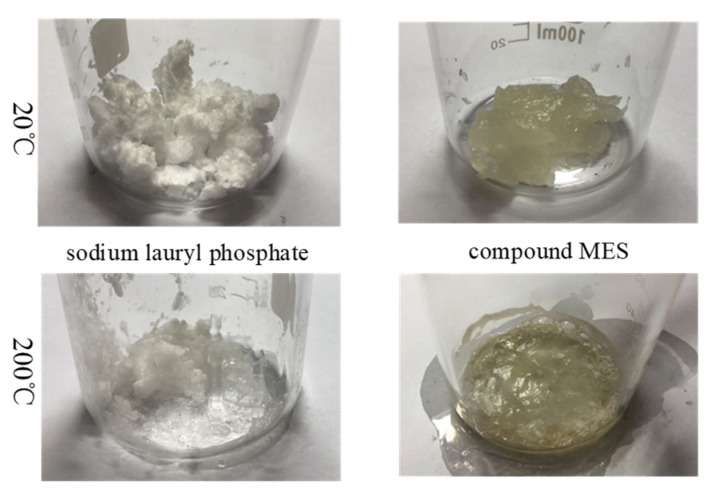
Comparison of apparent properties of surfactants combination B at 20 °C and 200 °C.

Surfactants are employed to maintain the sedimentation stability of MR fluids. When formulating an MR fluid with high-temperature and shear-thinning resistance, it is essential to ensure that the surfactants do not degrade at elevated temperatures in order to preserve effective sedimentation stability. In a testing environment with a maximum temperature of 200 °C, the color, odor, and volume of each surfactant in combinations A and B were individually evaluated and recorded. These properties were also compared with those observed at room temperature, as illustrated in Figure 4 and Figure 5.

As shown in Figure 4, for each reagent in combination A: water-soluble imidazoline appears as an amber-colored liquid at room temperature and remains in liquid form after heating, with only minor evaporation observed. Ethylene glycol monostearate exists as white flake-like particles at room temperature; it melts into a translucent liquid upon heating and recrystallizes into a white solid upon cooling. Glyceryl monostearate is a white powder at room temperature, which melts into a light-yellow liquid when heated and solidifies back into a white solid after cooling. The silane coupling agent is a transparent liquid at room temperature and retains its transparency and liquid state after heating without noticeable evaporation. Hydrophobic fumed silica presents as a light white powder with low density at room temperature and maintains its powdered form after heating.

As shown in Figure 5, for each reagent in combination B: sodium lauryl phosphate appears as a white paste at room temperature and partially melts upon heating, accompanied by a reduction in volume and an increase in density. Additionally, white smoke is observed during the heating process. The compound MES exists as a light yellow gel at room temperature; it transitions into a light yellow liquid when heated and solidifies into a light yellow gel-like substance upon cooling. Prolonged heating causes it to transform into a gray-black liquid.

Experimental results indicate that the sedimentation rate of combination B (1.61%) is significantly lower than that of combination A (6.12%), which demonstrates its superior ability to suppress particle settling. During high-temperature testing, water-soluble imidazoline in combination A exhibited evaporation, leading to reduced stability under prolonged exposure to elevated temperatures. Furthermore, the nonionic surfactants in combination B (glyceryl monostearate) show better compatibility with dimethyl silicone oil, enabling uniform dispersion within the oil phase and preventing performance inconsistencies caused by localized aggregation.

### 3.3. Base Carrier Fluid

(1) Expansion characteristic

**Figure 6:**
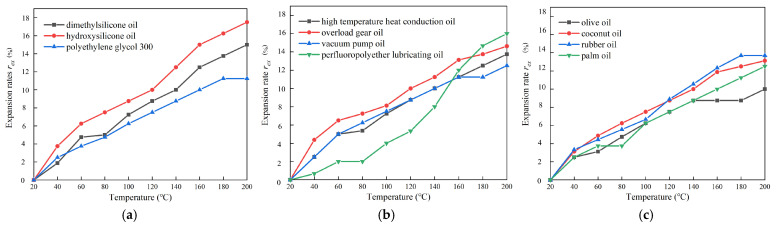
Expansion rates of base carrier fluids at different temperatures: (**a**) polymer oil; (**b**) synthetic oil; (**c**) natural oil.

When MR fluid operates in a high-temperature environment, the amplitude of particle vibration increases with rising temperature, leading to volumetric expansion of the MR fluid. Significant expansion can exert pressure on other components within MR devices, potentially causing mechanical deformation and MR fluid leakage. The base carrier fluid typically constitutes more than 70% of the MR fluid by volume, and its thermal expansibility significantly influences the overall expansion behavior of the MR fluid. To evaluate this characteristic, the base carrier fluid was heated from 20 °C to 200 °C, and its volume was recorded at 20 °C intervals. The expansion rate, *r_ex_*, was then calculated using Equation (2).

(2) $$r_{ex}=\frac{V_{rex}}{V_{o}}\times 100\%$$

where *V_rex_* is the recorded volume after the heat, and *V_o_* is the original volume before heating. The expansion-temperature curves of the eleven kinds of base carrier fluids are shown in Figure 6.

As can be seen from Figure 6, the expansion rate of the three types of oil-based liquids increases gradually with the temperature rising, in which the expansion rate of polymer oil and synthetic oil is higher, while the expansion rate of natural oil is lower. Among the three polymer oils, the expansion rate of hydroxy-silicone oil is the highest (16.0%), and that of polyethylene glycol 300 is the lowest (11.3%). Among the four synthetic oils, the expansion rate of perfluoropolyether lubricating oil is the highest (16.0%), and that of vacuum pump oil is the lowest (12.5%). Among the four natural oils, the expansion rate of rubber oil is the highest (13.7%), and the expansion rate of olive oil is the lowest (10.0%). The expansion rate of the most commonly used dimethyl silicone oil is 15%. Among the 11 kinds of oil-based carrier fluids proposed above, except for hydroxyl silicone oil and perfluoropolyether lubricant, the expansion rate of the 8 kinds of oil-based carrier fluids is lower than that of dimethyl silicone oil, with good high-temperature stability. At temperatures below 150 °C, the expansion rate of perfluorinated polyether lubricating oil is lower than that of other 10 oil-based carrier fluids, so its other characteristics can be further explored.

(2) Evaporation characteristic

**Figure 7:**
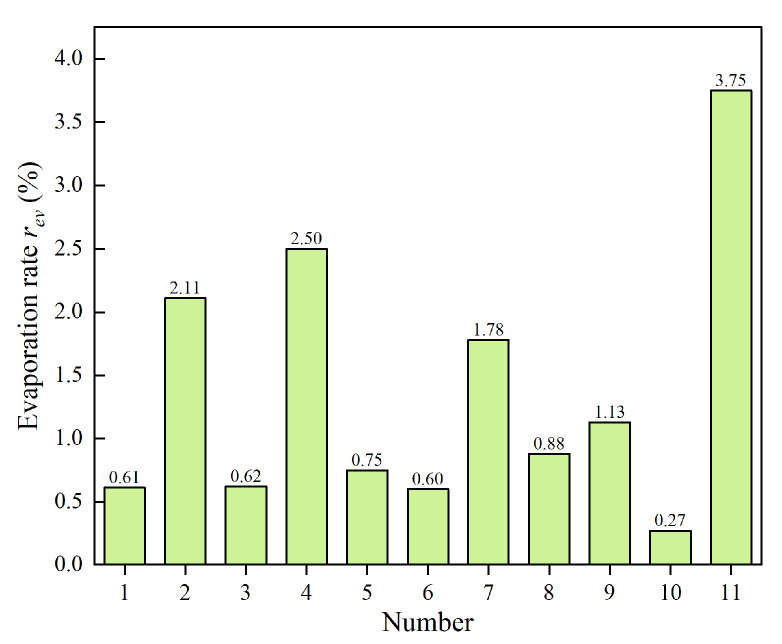
Evaporation rates of base carrier fluids at high temperatures.

As temperature increases, the Brownian motion of particles in the magnetorheological fluid becomes more intense. When this motion reaches a critical threshold, particles overcome interparticle forces and escape, resulting in a macroscopic phase transition from liquid to gas. Similarly, the base carrier liquid typically constitutes more than 70% of the magnetorheological fluid by volume, making it essential to evaluate its evaporation characteristics. Eleven types of base carrier liquids listed in Table 4 were placed in a high-temperature drying oven, heated to 200 °C, and maintained at this temperature for two hours. After cooling to room temperature, the remaining volume of each base carrier liquid was measured, and the evaporation rate, *r_ev_*, at different temperatures was calculated using Equation (3).

(3) $$r_{ev}=\frac{V_{rev}}{V_{o}}\times 100\%$$

where *V_rev_* is the recorded volume after the heat, and *V_o_* is the original volume before the heat. Figure 7 shows the evaporation rates of eleven kinds of base carrier fluids rev at high temperatures.

As shown in Figure 7, evaporation occurs in all eleven types of base carrier fluids after two hours of heating. Rubber oil exhibits the lowest evaporation rate (0.27%), whereas palm oil has the highest (3.75%). The evaporation rates of hydroxyl silicone oil, high-temperature heat conduction oil, perfluoroether lubricating oil, and palm oil exceed 1%. Repeated heating leads to a decrease in MR fluid volume and changes in particle concentration, which compromise the reliability of MR devices. Therefore, these fluids are not suitable as base carrier fluids for MR fluids. The remaining seven oil-based carrier fluids have evaporation rates below 1%, with polyethylene glycol 300 and rubber oil showing lower evaporation rates than dimethyl silicone oil, indicating superior high-temperature stability.

In addition, some base carrier fluids exhibit color changes after two hours of heating. Polyethylene glycol 300 changes from colorless to brown, high-temperature thermal oil changes from colorless to light yellow, and both rubber oil and palm oil also turn light yellow. These color changes suggest that these four base carrier fluids undergo structural or chemical alterations at elevated temperatures.

(3) Viscosity

**Figure 8:**
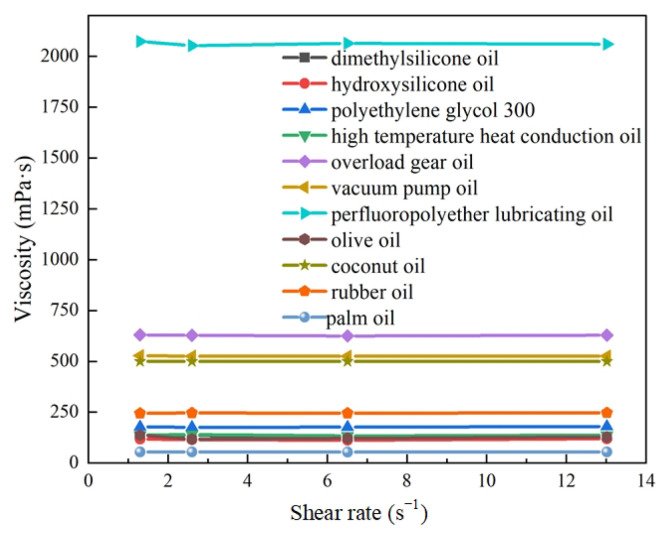
Viscosities of base carrier fluids at different shear rates and room temperature.

The zero-field fluidity of MR fluid is primarily determined by the viscosity of the base carrier fluid, which also plays a critical role in sedimentation stability. Previous studies [29] have demonstrated that the viscosity of base carrier fluids varies significantly with temperature; therefore, it is essential to investigate viscosity across different temperature conditions. Since carrier fluids can be classified as Newtonian or non-Newtonian, the Newtonian behavior of oil-based carrier fluids was first examined. Based on the characteristics of these fluids, an SNB-1 digital rotational viscometer (parameters listed in Table 5) was employed to measure the viscosities of the eleven selected oil-based carrier fluids at shear rates of 1.3 s^−1^, 2.6 s^−1^, 6.5 s^−1^, and 13.0 s^−1^. Figure 8 presents the viscosities of these eleven base carrier fluids under varying shear rates. All experiments were conducted at room temperature.

**Figure 9:**
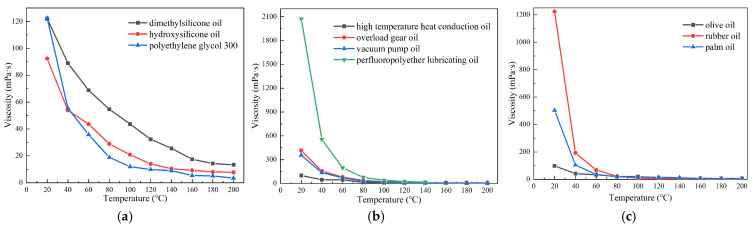
Viscosity-temperature curves of base carrier fluids at the shear rate of 13 s^−1^: (**a**) polymer oil; (**b**) synthetic oil; (**c**) natural oil.

As shown in Figure 8, the viscosity of base carrier fluids is almost constant at different shear rates, indicating that the above 11 base carrier fluids are all Newtonian fluids. Therefore, the viscosity at any shear rate can be selected to represent the viscosity of the base carrier fluid at the temperature. In the temperature range from 20 °C to 200 °C, the viscosities of the eleven kinds of base carrier fluids were measured with 20 °C as the temperature gradient, as shown in Figure 9. The experiments were carried out at a shear rate of 13 s^−1^. As the coconut oil is solid at room temperature, its viscosity data is missing.

As shown in Figure 9, the viscosity of each base carrier fluid gradually decreases with increasing temperature. The trend can be described as follows: the viscosity drops rapidly when the temperature rises from 20 °C to 80 °C, and reaches a lower level at 160 °C. As the temperature continues to increase beyond this point, the viscosity remains low and only slightly decreases further. Since viscosity originates from intermolecular forces, the increase in intramolecular energy with rising temperature weakens these forces, resulting in a reduction in viscosity at the macroscopic level.

At room temperature, perfluoropolyether lubricating oil and rubber oil exhibit significantly higher viscosities, with perfluoropolyether lubricating oil exceeding 2 Pa·s—close to the viscosity of typical commercial MR fluids. When magnetic particles are dispersed in such high-viscosity base carrier fluids, the viscosity of the suspension increases substantially, which may impair the fluidity of the MR fluid. Consequently, perfluoropolyether lubricating oil and rubber oil are not suitable as base carrier fluids for MR fluids. Although overload gear oil and vacuum pump oil also show relatively high viscosities at room temperature, their values remain within an acceptable range, making them candidates for further evaluation of other performance properties.

(4) Shear-thinning resistance

**Figure 10:**
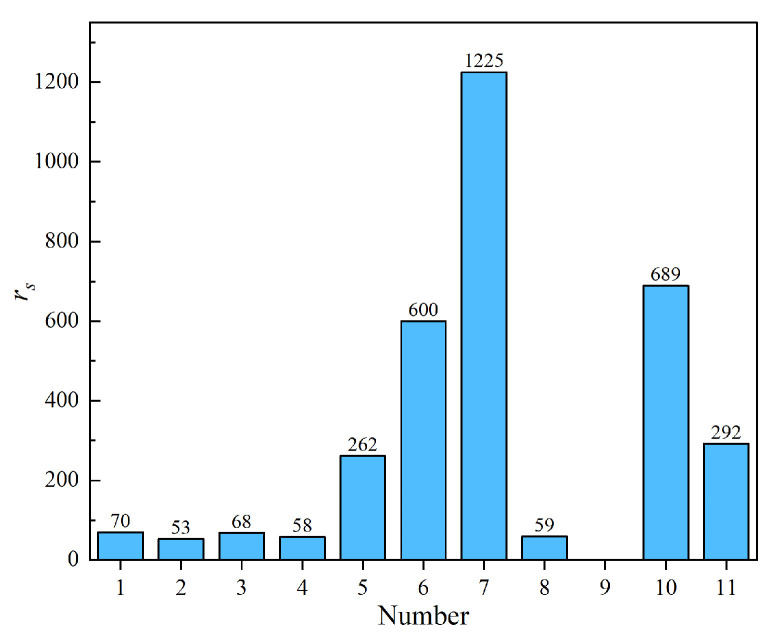
Comparison of shear-thinning effects of MR fluids prepared with different base carrier fluids.

To investigate the anti-shear thinning effect of base carrier fluid, the MCR 302 rotary rheometer and MRD 170/1 T rheological module by ANTON PAAR respectively obtained the apparent viscosity of MR fluids prepared by eleven kinds of base carrier fluids at different rates under the magnetic field of 817 mT, and calculated the ratio rs of the maximum apparent viscosity *η*_max_ and the minimum apparent viscosity *η*_min_ based on Equation (1), as shown in Figure 10. The flake-shaped powder is used to prepare the MR fluid, and the experiments were carried out at room temperature.

As shown in Figure 10, the shear-thinning behavior of MR fluids prepared with different base carrier fluids varies significantly under a magnetic field of 817 mT. A comparison of Figure 9 and Figure 10 reveals that the shear-thinning effect of the MR fluid is negatively correlated with the room-temperature viscosity of the base carrier fluid; higher room-temperature viscosity leads to a more pronounced shear-thinning effect. Among the eleven candidate base carrier fluids evaluated, perfluoropolyether lubricating oil, vacuum pump oil, rubber oil, palm oil, and overload gear oil exhibit a ratio of maximum apparent viscosity to minimum apparent viscosity exceeding 200. This large ratio indicates severe shear thinning during shearing under a magnetic field, making these fluids unsuitable as base carrier fluids for MR fluids. In contrast, dimethyl silicone oil, hydroxyl silicone oil, polyethylene glycol 300, high-temperature heat transfer oil, and olive oil show relatively low viscosity ratios and demonstrate good resistance to shear thinning, making them suitable candidates for use as base carrier fluids.

(5) Suitable base carrier fluid

Table 5 compares the expansion rate, evaporation rate, viscosity-temperature characteristics, and shear-thinning resistance characteristics of eleven kinds of base carrier fluids at high temperatures. In Table 6, “√” indicates that the liquid has a good performance and is suitable to be used as the base carrier fluid. “×” indicates that the liquid has a poor performance and is not suitable for the base carrier fluid of MR fluid.

As shown in Table 6, based on experimental results regarding the expansion rate, evaporation rate, sedimentation rate, and shear-thinning resistance of base carrier fluids, dimethyl silicone oil, polyethylene glycol 300, and olive oil exhibit favorable apparent and mechanical properties, making them suitable candidates for use as base carrier fluids in MR fluids with high-temperature and shear-thinning resistance.

Although polyethylene glycol 300 and olive oil demonstrate excellent performance in certain properties, such as low evaporation and expansion rates, polyethylene glycol 300 is prone to discoloration and thermal degradation at elevated temperatures. Olive oil, being a natural product, is susceptible to oxidation and lacks long-term thermal stability. In contrast, dimethyl silicone oil not only exhibits a low high-temperature evaporation rate (0.8%) and an acceptable expansion rate (15%), but also effectively mitigates shear thinning. Furthermore, its chemical inertness, well-established industrial application, and excellent compatibility with soft magnetic particles make it the most reliable base carrier fluid. This combination of properties ensures the long-term stability of MR fluids under high-temperature conditions.

## 4. Preparation of MR Fluid

**Figure 11:**
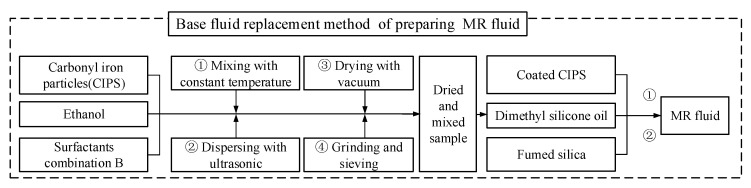
Base fluid replacement of preparing MR fluid.

The novel MR fluid with high-temperature stability and shear-thinning resistance was prepared using the base fluid replacement method [28]. This method is a technique that enhances the performance of MR fluids by substituting the original base fluid during the preparation process. Typically, MR fluids consist of magnetic particles (such as carbonyl iron particles, CIPs) dispersed in a base fluid (e.g., silicone oil or water). However, the original base fluid may exhibit unsuitable viscosity, poor sedimentation stability, or inadequate thermal stability. Compared to the traditional preparation method, the base fluid replacement method includes an additional step of coating magnetic particles with surfactants, allowing for more effective surfactant coverage. This improvement significantly enhances the stability of the resulting MR fluid. The detailed preparation process of the base fluid replacement method is illustrated in Figure 11.

## 5. Performance of MR Fluid

Based on the above research, the novel MR fluid was prepared, and the apparent and mechanical performance were tested. Besides, the performance of the novel MR fluid was also compared with the commercial MRF-J25T (Chongqing Materials Research Institute Co., Ltd., Chongqing, China). Table 7 exhibits the information for MRF-J25T.

### 5.1. Apparent Performance

(1) Sedimentation rate

**Figure 12:**
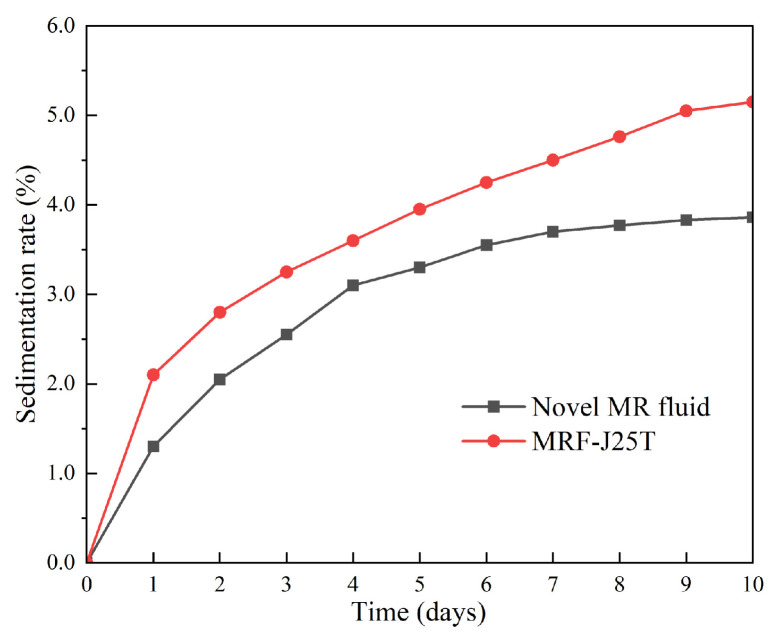
Sedimentation rate of novel MR fluid.

To evaluate the sedimentation stability of the novel MR fluid, the prepared sample was placed in a measurement cylinder and left undisturbed. The volume of the precipitated supernatant was recorded daily. The sedimentation observation method was employed to calculate the sedimentation rate of both the novel MR fluid and MRF-J25T, as illustrated in Figure 12.

As can be seen from Figure 12, the sedimentation rate of the MR fluid gradually increases with standing time; however, the rate of increase begins to slow after the 4th day. The soft magnetic particles in the MR fluid attract and collide with each other under the influence of van der Waals forces, leading to cluster formation. Due to the significant density difference between these particle clusters and the base carrier fluid, sedimentation occurs. As some particles aggregate and settle, the average distance between remaining particles increases, resulting in a reduction in van der Waals forces and a gradual slowing of the sedimentation process [37].

The sedimentation rate of the novel MR fluid reaches 3.72% after one week of standing and increases to 3.86% after 10 days. Throughout this period, the sedimentation rate of the novel MR fluid remains consistently lower than that of the commercial product, demonstrating its superior sedimentation stability. This ensures that when the novel MR fluid is applied in MR devices, sedimentation-induced failure will not occur.

(2) Expansion rate

**Figure 13:**
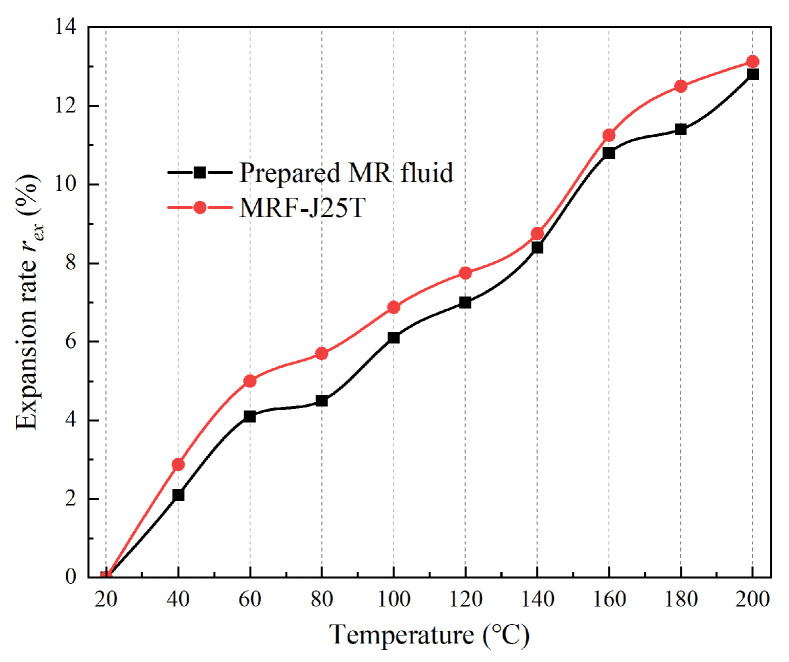
The expansion rate of novel MR fluid and MRF-J25T.

To determine the expansion characteristics of the MR fluid, the volume of the novel MR fluid was measured across a temperature range of 20 °C to 200 °C following the method described in Section 3.3. The expansion rate *r*_ex_ of both the novel MR fluid and the commercial fluid MRF-J25T at different temperatures was then calculated using Equation (2), as illustrated in Figure 13.

As can be seen from Figure 13, the expansion rate of the prepared sample almost increases linearly with temperature. Compared with Figure 6, the expansion rate of the prepared sample is lower than that of the base carrier fluid at the same temperature, because the expansion of MR fluid at high temperatures comes from the increase in the volume of base carrier fluid, while the soft magnetic particles with high Curie point will not expand in the temperature range from 20 °C to 200 °C. Therefore, it can be predicted that the expansion rate of MR fluid will gradually decrease with the increase in particle concentration. In the high-temperature environment of 200 °C, the expansion rate of the prepared sample is 2.8%, which is lower than that of the commercial MR fluid MRF-J25T, as also shown in Figure 13. The temperature stability of the novel MR fluid is good, and it will not cause the deformation of the components in MR devices or leakage of MR fluid.

(3) Evaporation rate

**Figure 14:**
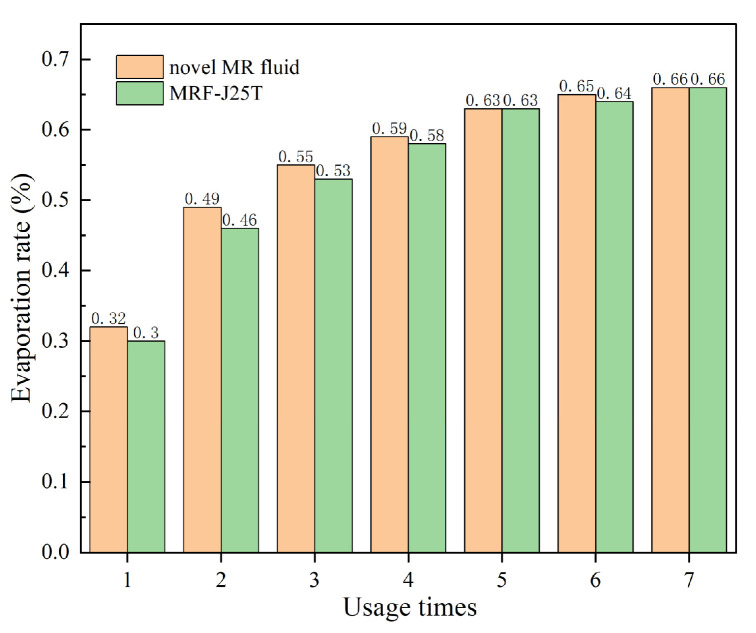
The evaporation rate of novel MR fluid and MRF-J25T.

To obtain the evaporation characteristics of the novel MR fluid, the sample was heated to 200 °C and kept for 2 h in the high-temperature oven. After cooling to room temperature, the volume of the sample was measured and recorded. The experiments were repeated 7 times. The evaporation rate *r*_ev_ of the MR fluid after repeated use is calculated by Equation (3), as shown in Figure 14.

As shown in Figure 14, after multiple heating cycles, the evaporation of the prepared MR fluid sample initially increases and then stabilizes. Compared with the data in Figure 7, the evaporation rate of the MR fluid is lower than that of the base carrier fluid alone. This is because the evaporation of MR fluid at high temperatures mainly results from the evaporation of the base carrier fluid, while the soft magnetic particles do not evaporate at 200 °C. The novel MR fluid exhibits an evaporation rate of only 0.66%, demonstrating excellent thermal stability. Although the novel MR fluid contains a higher proportion of base carrier fluid, leading to a slightly higher evaporation rate compared to MRF-J25T, it still maintains good thermal stability. This ensures that the mass fraction of the MR fluid remains nearly unchanged, preserving the operational stability of the MR device.

### 5.2. Mechanical Performance

(1) Viscosity

**Figure 15:**
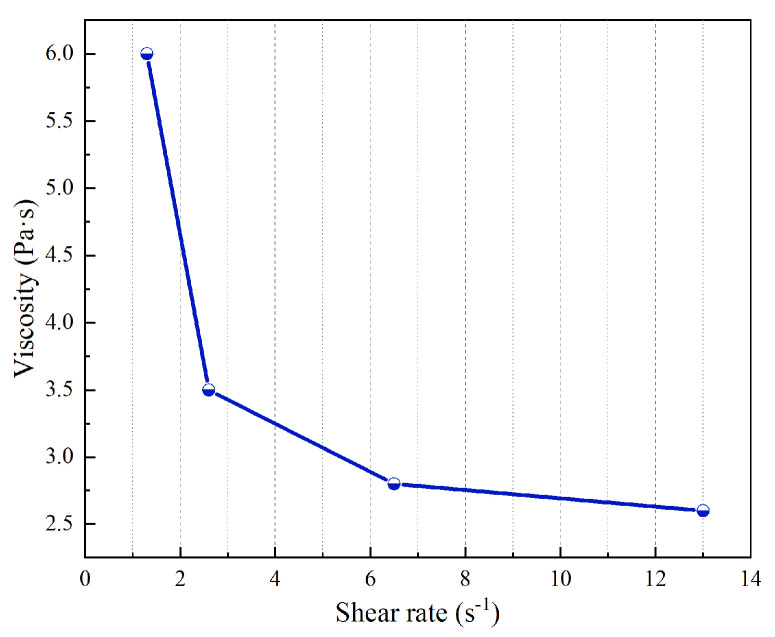
Viscosity of novel MR fluid.

The SNB-1 digital display rotational viscometer was used to obtain the viscosity of the MR fluid with high-temperature and shear-shinning resistance at 1.3 s^−1^, 2.6 s^−1^, 6.5 s^−1,^ and 13.0 s^−1^, respectively. The viscosity was measured in the absence of a magnetic field and at room temperature, as shown in Figure 15.

Figure 15 shows that the zero-field viscosity of the prepared MR fluid decreases rapidly with increasing shear rate. At rest, the randomly distributed magnetic particles form a three-dimensional network structure, which results in higher viscosity. When shear is applied, hydrodynamic forces progressively disrupt this network structure while aligning the particles along the flow direction. These combined effects contribute to the observed decrease in viscosity as the shear rate increases. It should be noted that although shear thinning occurs in the zero-field state, MR fluids typically operate under an applied magnetic field. Moreover, the shear-induced stirring in the zero-field state helps disperse the particles more uniformly, thereby inhibiting sedimentation. Although the viscosity of the prepared MR fluid is 6 Pa·s, which is slightly higher than the 1.45 Pa·s of MRF-J25T, as the shear rate increases, the viscosity of the prepared MR fluid will drop rapidly, indicating that its fluidity is relatively good and it is convenient to inject into the magnetorheological device.

(2) Yield stress

**Figure 16:**
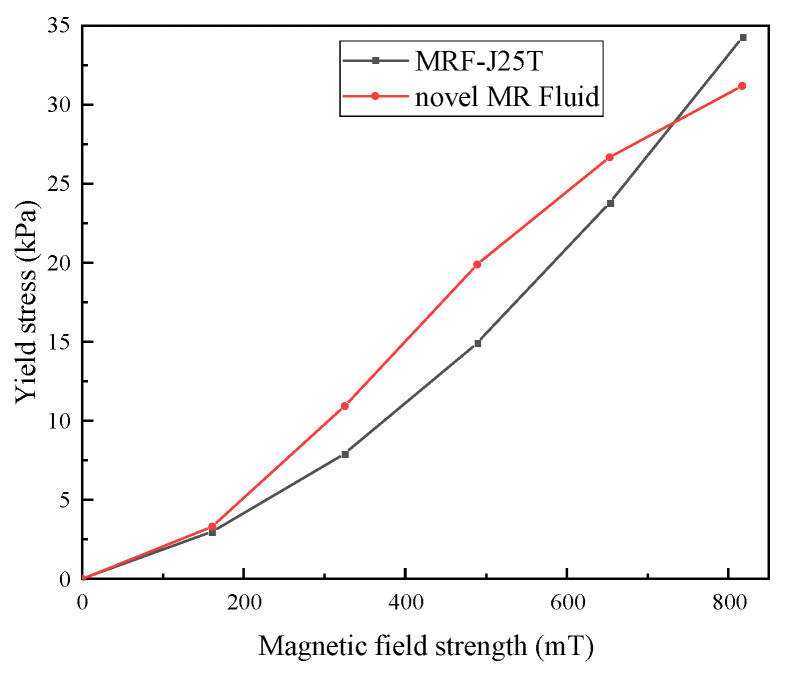
Shear yield stress of novel MR fluid.

The yield stress of the prepared MR fluid was measured under magnetic fields of 161 mT, 325 mT, 489 mT, 653 mT, and 817 mT using an MCR 302 rotational rheometer equipped with an MRD 170/1 T module (Anton Paar, Graz, Austria), as illustrated in Figure 16.

As shown in Figure 16, the shear yield stress of the prepared MR fluid increases rapidly with the magnetic field strength initially, after which the growth rate gradually slows down, eventually approaching a nearly constant value. This variation trend is consistent with the magnetization behavior of CIPs under different magnetic fields. The shear yield stress originates from the component of the interparticle magnetic force in the shear direction, and this magnetic force is induced by the magnetization of particles under an applied magnetic field. Therefore, the trend in shear yield stress corresponds to the magnetization trend of the particles. Under low magnetic field conditions, the yield shear stress of the prepared magnetorheological fluid is slightly higher than that of the MRF-J25T; while under high magnetic field conditions, the yield shear stress of MRF-J25T is relatively higher, and the gap tends to be significant. Under a magnetic field strength of 817 mT, the shear yield stress of the novel MR fluid with high-temperature and shear-thinning resistance reaches 31.2 kPa, indicating superior mechanical performance.

Based on the above theoretical analysis and experimental tests, the prepared MR fluid demonstrates significantly improved sedimentation stability and high temperature stability compared to MRF-J25T. Although its viscosity is slightly higher than that of MRF-J25T, the fluid still maintains good flow ability. The shear yield stress is notably increased compared to previous studies [1]. Under a magnetic field of 817 mT or lower, its performance is comparable to that of MRF-J25T; however, under high magnetic field conditions, its performance is slightly inferior.

Overall, the MR fluid developed in this paper exhibits excellent sedimentation stability, high temperature stability, and resistance to shear thinning, making it more suitable for MR devices operating under high temperature conditions.

## 6. Conclusions

To enhance the high-temperature stability and shear-thinning resistance of magnetorheological fluids, this study systematically investigates the performance of magnetic particles, surfactants, and base carrier fluids through experimental analysis. Suitable components are selected based on the results, leading to the successful preparation of a novel magnetorheological fluid with improved resistance to both high temperature and shear thinning. The specific research findings are summarized as follows:

(a) Compared with spherical CIPs, the flake-shaped CIPs exhibit superior shear-thinning resistance and higher yield stress, making them more suitable as soft magnetic particles for MR fluid. A surfactant combination consisting of sodium lauryl phosphate, compound MES, glycol monostearate, glycerol monostearate, and hydrophobic fumed silica effectively enhances the sedimentation stability and high-temperature stability of the MR fluid, thus being appropriate for MR fluids with high-temperature and shear-thinning resistance. Dimethyl silicone oil, which has a low expansion rate, low evaporation rate, suitable viscosity, and good shear-thinning resistance, is well-suited as the base carrier fluid.
(b) The MR fluid with high-temperature stability and shear-thinning resistance was prepared using the base fluid replacement method, and its performance was systematically evaluated. Results indicated that after standing for 10 days, the sedimentation rate of the prepared MR fluid is 3.86%. At 200 °C, the expansion rate reaches 12.8%, while the evaporation rate remains low at 0.66% after multiple heating cycles, demonstrating excellent high-temperature stability.
(c) The prepared MR fluid exhibits good fluidity at room temperature, with a viscosity of 6 Pa·s. Under a magnetic field of 817 mT, the shear yield stress of the prepared MR fluid is measured at 31.2 kPa. These properties collectively indicate that the MR fluid with high-temperature stability and shear-thinning resistance is a highly suitable medium for MR devices.

MR fluids exhibiting sedimentation stability, high-temperature stability, and resistance to shear thinning hold significant promise for applications in fields such as power transmission, vibration control, and precision polishing. Future research will focus on exploring the practical application of the prepared MR fluid in MR devices, along with a more comprehensive evaluation of its performance and functional characteristics.

## Figures and Tables

**Table 1:** Parameters of CIPs in different shapes.

Shape	Fe	C	O	D50/μm	Loose Density/g·cm^−3^	Vibration Density/g·cm^−3^	Mean Diameter/μm
ball	98.05%	0.84%	0.64%	1.92	2.20	4.08	2.00
flake	98.10%	0.69%	0.23%	4.09	2.80	4.20	3.70

**Table 2:** Surfactant combination A for MR fluid.

Component	Type	Main Component
water-soluble imidazoline	anionic surfactants	C_22_H_42_N_2_O
glycol monostearate	non-ionic surfactants	C_20_H_40_O_3_
glycerol monostearate		C_21_H_42_O_4_
Silane	thixotropic agent	H_2_N(CH_2_)_3_Si(OC_2_H_5_)_3_
hydrophobic fumed silica	thixotropic agent	SiO_2_

**Table 3:** Surfactant combination B for MR fluid.

Component	Type	Main Component
sodium lauryl phosphate	cationic surfactants	C_12_H_25_OPO_3_Na_2_
compound MES		RCH(SO_3_M)COOCH_3_
glycol monostearate	non-ionic surfactants	C_20_H_40_O_3_
glycerol monostearate		C_21_H_42_O_4_
hydrophobic fumed silica	thixotropic agent	SiO_2_

**Table 4:** Base carrier fluid for MR fluid.

Base Carrier Fluid	Main Component	Type
dimethyl silicone oil	polydimethylsiloxane	polymer oil
hydroxysilicone oil	siloxanes and polysiloxanes	
polyethylene glycol 300	polyethylene oxide	
high-temperature heat conduction oil	aromatics	synthetic oil
overload gear oil	mineral synthetic oil	
vacuum pump oil	hydrocarbon	
perfluoropolyether lubricating oil	perfluoropolyether	
olive oil	saponifiable and unsaponifiable	natural oil
coconut oil	lauric acid	
rubber oil	naphthenic oil	
palm oil	retinoic acid	

**Table 5:** Parameters of rotational viscometer SNB-1.

Brand	Measurement Range/Pa·s	Rotation Speed/r/min	Measurement Error	Repeatability Error
DECCA	20–600,000	1–60	±1%	±0.5%

**Table 6:** Performance parameters comparison of candidate base carrier fluids.

Base Carrier Fluid	Expansion Characteristic	Evaporation Characteristic	Viscosity	Shear-Thinning Resistance
dimethyl silicone oil	√	√	√	√
hydroxysilicone oil	√	×	√	√
polyethylene glycol 300	√	√	√	√
high-temperature heat conduction oil	√	×	√	√
overload gear oil	√	√	√	×
vacuum pump oil	√	√	√	×
perfluoropolyether lubricating oil	√	×	×	×
olive oil	√	√	√	√
coconut oil	√	√	×	×
rubber oil	√	√	×	×
palm oil	√	×	×	×

**Table 7:** Parameters of MRF-J25T.

Volume Fraction	Density/g/mL	Service Temperature/°C
25 vol%	2.65	±1%

## Data Availability

The original contributions presented in this study are included in the article. Further inquiries can be directed to the corresponding authors.
